# PAOT-Liquid^®^ Technology: An Easy Electrochemical Method for Evaluating Antioxidant Capacity of Wines

**DOI:** 10.3390/diseases7010010

**Published:** 2019-01-21

**Authors:** Pincemail Joël, Kaci Mouna-Messaouda, Kevers Claire, Tabart Jessica, Ebabe Elle Raymond, Meziane Smail

**Affiliations:** 1 Department of Cardiovascular Surgery/Antioxidant Nutrition and Health Platform, University of Liège and CHU, Sart Tilman, 4000 Liège, Belgium; raymond.elle@yahoo.fr; 2 Institute Européen des Antioxydants, University of Nancy, 18 rue Victor de Lespinats, 54230 Neuves-Maisons, France; mkaci@ie-antioxydants.com (K.M.-M.); smeziane@ie-antioxydants.com (M.S.); 3 Plant Molecular Biology and Biotechnology, University of Liège, Sart Tilman, 4000 Liège, Belgium; c.kevers@ulg.ac.be (K.C.); jessica.tabart@alumni.uliege.be (T.J.)

**Keywords:** polyphenols, antioxidant capacity, electrochemical technology, wine

## Abstract

Polyphenol compounds present in high quantity in wines are well-known to have potent cardio-protective properties through several biological mechanisms including antioxidant activity. A large number of methods have been developed for evaluating the antioxidant capacity of food matrices. Most of them have, however, the disadvantage of being time consuming and require specific analytical protocols and devices. In the present study, we present the electrochemical PAOT (Pouvoir Antioxydant Total)-Liquid^®^ Technology which can be easily used by winemakers for evaluating antioxidant activity of wine during all steps of making process. The methodology is based on the measurement of electric potential variation resulting from chemical reactions between wine polyphenols and a free radical mediator M^•^ as source of oxidants. Total antioxidant activity as estimated by the PAOT-Liquid^®^ activity was 6.8 fold higher in red wines (*n* = 14) when compared to rosé (*n* = 3) and white (*n* = 3) wines bought in a commercial market. Moreover, PAOT-Liquid^®^ activity was highly correlated with total polyphenols content (TPC) of all wines (r = 0.9540, *p* < 0.0001) and the classical DPPH (2,2-diphenyl-1-picryhydrazyl) assay which is often used for evaluating antioxidant capacity of food matrices (r = 0.9102, *p* < 0.0001).

## 1. Introduction

A large number of studies have evidenced that oxidative stress plays a key role in the development of several pathologies including cardiovascular, neurological and inflammatory diseases, cancer and diabetes [1]. Jones has defined oxidative stress as an imbalance between reactive oxygen species or ROS (including free radical and non-free radical species) and antioxidants in favor of the formers, leading to a disruption of the redox signaling and/or molecular damage to lipids, proteins and DNA [2]. Among antioxidants, a large amount of interest has been given to the large family of polyphenols which can be divided into lignans, stilbenes, tannins, phenolic acids (benzoic and cinnamic acids derivatives) and flavonoids (flavonols, flavanones, flavones, flavanols or catechins, anthocyans and isoflavones). The potential health benefits of polyphenols were first highlighted by the Zutphen’s study, which evidenced an inverse relationship between intake in diet flavonoids and the risk of developing cardiovascular diseases [3]. Moreover, the adhesion to the Mediterranean diet known for its richness in polyphenols is well recognized to be a guarantee of good cardiovascular health [4,5]. The capacity of polyphenols to regulate the arterial blood pressure by maintaining a good endothelium health [6] but also their ability to stimulate genes coding for the expression of antioxidant enzymes through Keap1/NrF2/ARE activation [7] have, among other mechanisms, prime places for explaining such cardio-protective effects. 

Repartition of polyphenols in natural foods is as follows: fruits (41%), fresh vegetables (11%), dry vegetables (8%) and processed products such as fruit juices, cocoa, coffee, green tea, olive oil but also red wine (33%). Over the past decade, the health effects of moderate red wine consumption (125 mL glass) by reducing risk of developing cancer and cardiovascular diseases have been the matter of many studies (for a review see references [8,9]). However, the wine polyphenol composition and, therefore, its antioxidant capacity can be strongly affected by winemaking techniques and oenological practices [10]. In the present paper, we present the PAOT-Liquid^®^ technology which is able to measure the total antioxidant capacity of wine, and indirectly their total polyphenol content (TPC), thanks to a fast electrochemical application. 

## 2. Material and Methods

Antioxidants gallic acid (GA), catechin (C), epicatechin (EC), epigallocatechin gallate (EGCG), epigallocatechin (EGC), gallocatechin (GC), myricetin, quercetin, kaempherol, naringin, hesperdin methyl calcone, cyanidin chloride, delphinidin chloride, pelargordin chloride, free radical 2,2-diphenyl-1-picryhydrazyl (DPPH) and Trolox (T) were all purchased from Sigma, Nancy and Lyon, France. Folin’s reagent, methanol and sodium carbonate have been supplied by WWR International, Fontenay-sous-Bois, France. Wines including 14 red, 3 rosé and 3 white produced in five different countries have been bought in a commercial market in Belgium.

### 2.1. Total Polyphenols Content (TPC)

Total polyphenols content was determined by the Folin–Ciocalteu method [11]. Appropriately diluted extract (3.6 mL) was mixed with 0.2 mL Folin–Ciocalteu reagent and 3 min later, 0.8 mL sodium carbonate (20% *w*/*v*) was added. The mixture was heated at 100 °C for 1 min. After cooling, the absorbance at 750 nm was measured. Using gallic acid (GA) as a standard, results were expressed as mg gallic acid equivalents/par liter (GAE) L^−1^.

### 2.2. DPPH Assay

Antioxidant capacity of wines was determined by the DPPH (free radical 2,2-diphenyl-1-picryhydrazyl) assay as initially described by Tadolini et al. [12]. All complete details about the protocol were provided in a previous paper of us [13]. Trolox (T) was used as standard and the antioxidant capacity was expressed in µmol Trolox equivalent/liter (TE) L^−1^.

### 2.3. PAOT-Liquid^®^ Assay

**Figure 1:**
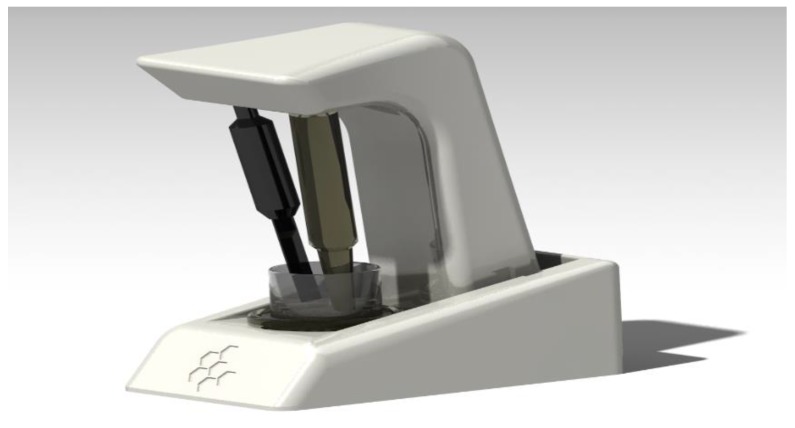
Photography of the PAOT-Liquid^®^ Technology device showing both reference and working microelectrodes immersed in the reaction medium containing free radical mediator M^•^ and antioxidants or wines samples.

**Figure 2:**
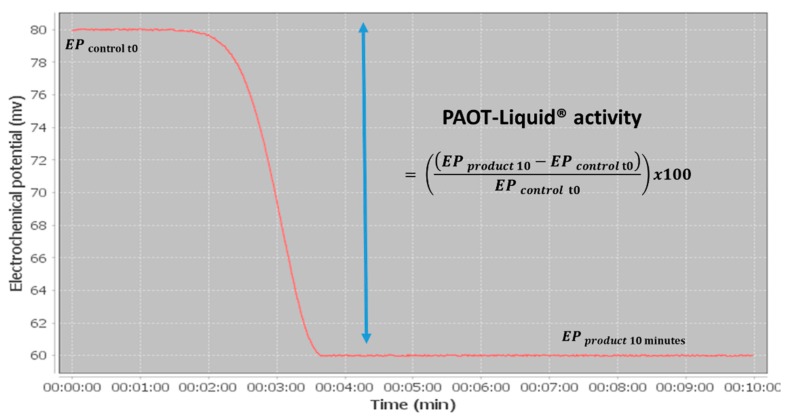
Kinetic curve of electrochemical potential changes during reaction of antioxidants or wines samples with the free radical mediator M^•^.

PAOT (Pouvoir Antioxydant Total) Liquid^®^ Technology is a method allowing total antioxidant capacity determination in various matrices, such as raw materials and processed food products, cosmetic and medicinal preparations, biological fluids or plant extracts [14]. The PAOT Liquid^®^ Technology is actually the subject of a patent application filing (patent FR1871986; 11.28.2018). Thanks to the robust and easily transportable device shown on Figure 1, the measurement was carried out in a reaction medium (1 mL physiological solution at pH ranging from 6.7 to 7.2, temperature 24–27 °C) containing a molecule in a free radical state called mediator (M^•^). Two microelectrodes, one being the working electrode and the second one the reference electrode, were then immersed in the medium. After addition of 20 μL of pure antioxidants (1 mM final) or wine samples, PAOT-liquid^®^ activity was estimated by registering electrochemical potential modifications in the reaction medium (due to changes in the concentration of oxidized/reduced forms of the mediator M^•^ during reaction with antioxidants as AOX (oxidized mediator M^•^ + AOX -→ reduced mediator M + oxidized AOX) [15]. Figure 2 shows the typical curve of the electrochemical potential registration after 10 min of interaction of AOX or wine simples with mediator M^•^. Results were calculated according to the following formula:

(1) $$\mathrm{antioxidant}\;\mathrm{activity}=\left(\frac{\left({EP}_{product\;10}-{EP}_{control\;0}\right)}{{EP}_{control\;0}}\right)\times 100\%,$$

where *EP_control_*_0_ was the electrochemical potential at time 0 and *EP_product_*_10_ the electrochemical potential obtained after 10 min registration in presence of tested antioxidants or wine samples. Gallic acid was used as a standard and results were expressed as mg gallic acid equivalents (GAE) L^−1^.

## 3. Results

Table 1 summarizes the characteristics of all tested wines (14 red, 3 rosé and 3 white) produced in different countries (France, Italy, South Africa, Chili and South Australia).

Table 2 describes the PAOT-Liquid^®^ activity of main polyphenols, more particularly those of the flavonoid family, which can be found in wines. Tested at a concentration of 1 mM, myricetin belonging to the flavonol family exhibited the highest PAOT-Liquid^®^ activity (677.78 mg (GAE) L^−1^) when compared to quercetin (560.4 mg (GAE) L^−1^) and kaempferol (404.56 mg (GAE) L^−1^). In the anthocyanins family, cyanidin had the best score (512.54 mg (GAE) L^−1^) in front of delphinidin and pelargordinin. Both EC (730.2 mg (GAE) L^−1^) and EGCG (613.11 mg (GAE) L^−1^) from the favano-3-ol subgroup were among all tested molecules those having the highest antioxidant capacity. For comparison, Trolox which is the antioxidant reference used in most in vitro assays, had only a value of 544.16 mg (GAE) L^−1^. At least, both naringin (53.28 mg (GAE) L^−1^) and hesperidin methyl calcone (51.85 mg (GAE) L^−1^) from the flavanone group presented a score which was largely below those of all other tested flavonoids.

As shown in Table 3, the highest TPC (mean value: 1789 ± 367 mg (GAE) L^−1^) was clearly found in red wines when compared to rosé (mean value: 265 ± 65 (GAE) L^−1^) and white (mean value: 221 ± 28 mg (GAE) L^−1^) wines. As suggested daily allowance in total polyphenols is around 1000 mg [16], the consumption of 125 mL glass of red wine, therefore, meanly affords 223 mg of TP. A large heterogeneity was, however, observed in red wines since values may vary from 1278 (wine 12) to 2349 mg (GAE) L^−1^ (wine 3). A total of 5/14 red wines had a TPC higher than 2000 mg (GAE) L^−1^ (wines 1, 3, 4, 10 and 13). Three of them (3, 4, 13) were multi-varietal while the two other ones were mono-varietal (1, 10). By contrast, 9/14 wines (2, 5, 6, 7, 8, 9, 11, 12, 14,) had values between 1278 and 2000 mg (GAE) L^−1^. Six of them (2, 6, 7, 11, 12, 14) were mono- or bi-varietal and three multi-varietal (5, 8, 9). Statistical analysis revealed, however, that there was not significant difference between the mean value in TPC of mono or bi and multi varietal wines (1733 ± 125.6 mg (GAE) L^−1^, *n* = 8 vs. 1864 ± 164.5 mg (GAE) L^−1^, *n* = 6; *p* = 0.57).

**Figure 3:**
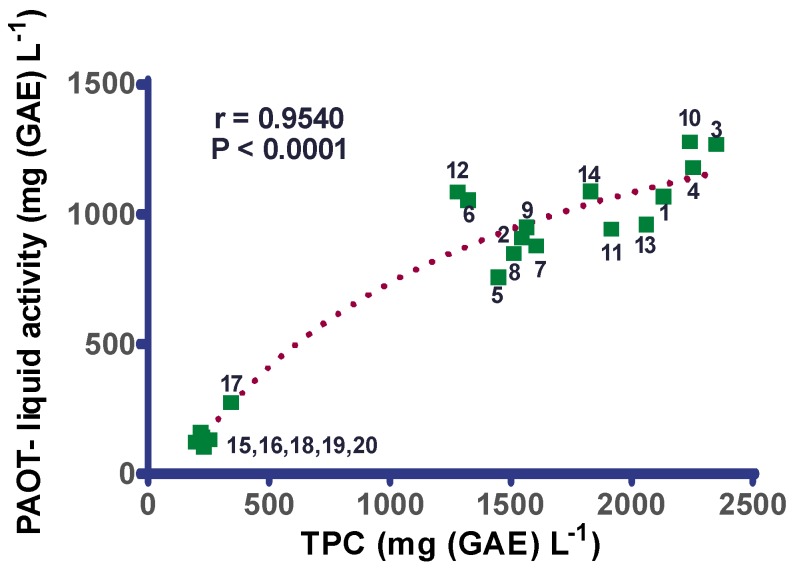
Correlation between TPC (total polyphenols content) and PAOT-Liquid^®^ activity in red (*n* = 14), rosé (*n* = 3) and white wines (*n* = 3) bought in a Belgian commercial market.

**Figure 4:**
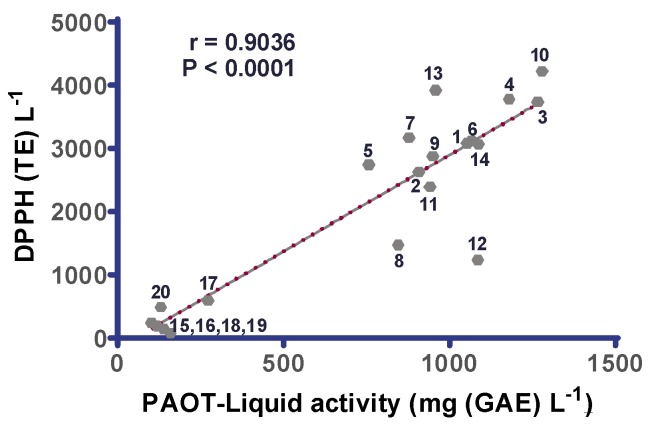
Correlation between PAOT-Liquid^®^ activity and DPPH assay in red (*n* = 14), rosé (*n* = 3) and white wines (*n* = 3) bought in a Belgian commercial market.

**Figure 5:**
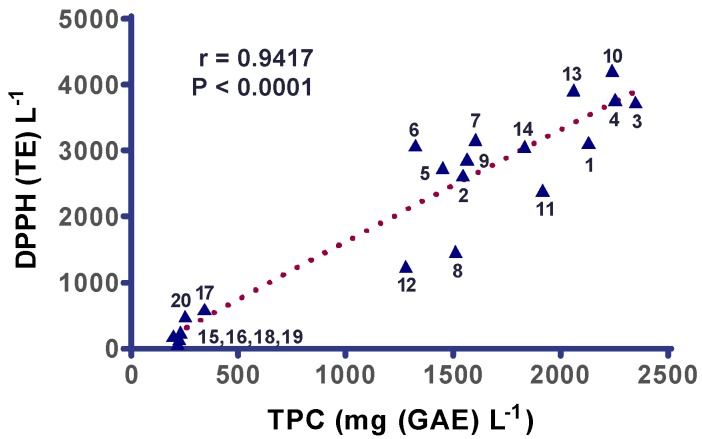
Correlation between TPC (total polyphenols content) and antioxidant capacity as assessed by DPPH assay in red (*n* = 14), rosé (*n* = 3) and white wines (*n* = 3) bought in a Belgian commercial market.

Figure 3 evidences that there was a strong positive and significant correlation (r = 0.9540, *p* < 0.0001) between TPC and PAOT-Liquid^®^ activity. The deep shift between red wines and rosé and white ones was confirmed. Among red wines, two different groups were identified as for TPC: wines 1, 3, 4, 10, 11, 13, 14 vs. wines 2, 5, 6, 7, 8, 9, 12. As shown on Figure 4 and Figure 5, similar correlations were also evidenced when comparing PAOT-Liquid^®^ activity and DPPH assay (r = 0.9036, *p* < 0.0001) or TPC and DPPH assay (r = 0.9417, *p* < 0.0001).

## 4. Discussion

A large number of methods have been developed to determine the in vitro antioxidant capacity of food matrices. They include two major groups: assays based on single electron transfer reaction (SET), in which the redox reaction between the antioxidant and the oxidant is measured by the change in the oxidant’s color, as an indicator of the end of the reaction; and assays based on hydrogen atom transfer reaction (HAT), in which there is a competitive reaction between the antioxidant and the substrate (probe) for the free radicals. SET methods are Trolox Equivalent Antioxidant Capacity (TEAC) assay, Ferric Reducing Ability (FRAP) assay, Copper Reduction (CUPRAC) assay, and, finally, 2,2-diphenyl-1-picrylhydrazyl radical scavenging capacity (DPPH) assay which is the most popular. HAT assays include the crocin bleaching assay, the total peroxyl radical trapping antioxidant parameter (TRAP) assay, and overall, the Oxygen Radical Absorbance Capacity (ORAC) assay. Advantages and disadvantages of all these methods have been discussed in detail in a previous paper of us [17]. The PAOT-Liquid^®^ Technology can be classified in the SET category since this electrochemical assay directly estimates the antioxidant capacity via the electric potential shift due to changes in the concentration of oxidized/reduced forms of the free radical mediator (M^•^) during reaction with antioxidants. Moreover, the use of microelectrodes for registering current changes from reaction between the oxidant mediator M^•^ and antioxidants rendered the method very sensitive.

As shown in Table 2, the PAOT-Liquid^®^ Technology was perfectly able to evaluate the antioxidant capacity of molecules present in wines such as polyphenols from the flavonoids family. The relationship between PAOT-Liquid^®^ activity and the structure of these compounds can be even evidenced. The basal chemical structure for all flavonoids is constituted of a benzene A ring linked to an oxidized heterocyclic C ring substituted in position 2 by another benzene B ring. In the flavanone family, two phenolic (–OH) groups are present on ring A in positions 5 and 7, one on the ring C in position 4 while ring B is respectively substituted by 1, 2 and 3 –OH groups respectively in case of kaempferol (position 4′), quercetin (positions 3′ and 4′) and myricetin (positions 3′, 4′ and 5′). Table 2 shows that the PAOT-Liquid^®^ activity logically increased with the number of antioxidant –OH groups on ring B, myricetin having so the highest value in front of quercetin and kaempferol.

In the flavanol-3-ol family, benzene A rings possess two –OH groups on positions 5 and 7 while one –OH group is present on the heterocycle C ring on position 3. Ring B is substituted with 2 –OH groups in case of catechin and its isomer epicatechin (EC) on positions 4′ and 5′. Gallocatechin (GC) and epigallocatechin (EGC) have another –OH group on position 3′. When compared to GC, the chemical structure of epigallocatechingallate (EGCG) has the –OH group on the heterocyclic C ring substituted by a gallate group constituted of a benzene ring having 3 –OH groups. Due its large number of –OH groups (*n* = 9), EGCG has, as expected, one of the highest PAOT-Liquid^®^ activity. It is instructive to note that both isomers of catechin and epicatechin have a higher antioxidant activity than the original form.

In the anthocyanins family, benzene ring A with two –OH groups on positions 5 and 7 is linked to a flavylium cation having a –OH group in position 3 and in position 2 by the benzene B ring. In case of pelagornidin, cyanidin and delphinidin, three of the six main anthocyanins present in red wine, B ring is respectively substituted by 1 (position 4′), 2 (positions 3′ and 4′) and 3 (3′, 4′ and 5′) –OH groups. According to its number of antioxidant –OH groups, pelagornidin has the lowest PAOT-Liquid^®^ activity when compared to delphinidin and cyanidin as shown in Table 2.

Molecules from the flavanone family are characterized by the presence of one –OH group on ring A (position 5) and another one on ring C (position 4′). In the case of naringin and hesperidin methyl calcone, the –OH group on ring B (position 7) is substituted by a rutinose moiety, resulting in an important loss in the antioxidant capacity.

Table 3 shows that there was a clear shift between red, rosé and white wines with respect to their TPC. Mean TPC for red wines was 1789 ± 367 mg (GAE) L^−1^ against only 265 ± 65 for rosé and 221 ± 28 for white wines. These results are in agreement with literature data [18,19]. Among tested red wines, a large heterogeneity in TPC was evidenced. Two groups of values have been observed, those above (*n* = 5) or below (*n* = 9) 2000 mg (GAE) L^−1^. However, we did not observe significant difference in TPC between red wines constituted of mono-, bi- or multi-varietals as also reported by Paixao et al. [19]. By contrast, a great homogeneity in low TPC was observed for rosé and white wines.

A shown in Figure 3, we evidenced that the PAOT-Liquid^®^ activity and TPC of wines were highly correlated (r = 0.9540; *p* < 0.0001). Other authors using electrochemical detection with laccase biosensor [18], poly(3,4-ethylenedioxythiophene)-modified electrodes [20] or carbon nanotube-modified electrodes [21] reported similar findings. The high correlation between TPC and PAOT-Liquid^®^ activity provided such strong evidence that the majority of the antioxidant activity was attributed to the polyphenolic compounds in such beverages. In a recent study [22], we concluded that the relative percentages of various classes of polyphenol compounds for red wines having only one grape variety (Merlot, Syrah, Cabernet Sauvignon) were as follows: 24.3% phenolic acids, 7.4% flavonols, 37.3% flavanols, 30.4% anthocyanidins and only 0.4% resveratrol (16). The grape variety Pinot Noir exhibited a different profile with less flavonols (2.8%) and anthocyanidins (14.6%), but more flavanols (54.9%).

Of interest was the evidence for a strong correlation between PAOT-Liquid^®^ activity and DPPH assay as shown in Figure 4. Even if the wine matrix is the same in both assays, we have chosen to express the results in two different antioxidant scales. Indeed, chemical and synthetic Trolox was conventionally used as reference antioxidant molecule in all papers referring to DPPH assay. By contrast, it was more logical to express antioxidant activity of wines evaluated by the PAOT-Liquid^®^ Technology by comparing to a natural antioxidant present in wine as it is the case for gallic acid. A great advantage of the PAOT-Liquid^®^ Technology is that there is no interaction between the color of wine and those developed during the reaction of DPPH with the samples. At least, correlation was found between TPC of wines and the classical DPPH assay (Figure 5), as expected [23].

## 5. Conclusions

In conclusion, we have developed the PAOT-Liquid^®^ Technology that turns out to be a direct and useful tool for evaluating antioxidant capacity of red, rosé and white wines. When compared to classical DPPH or ORAC assays which require long and fastidious protocols [24], the determination of antioxidant capacity of wines evaluated by the PAOT-Liquid^®^ Technology was achieved within 10 min and without requiring analytical systems such as spectrophotometers or plaque readers, rendering the method easily accessible to the winemaker himself. One of the great weakness of classical DPPH or ORAC assays for measuring antioxidant capacity is also the absence of standardized protocols. In the literature, there are substantial differences in sample preparation, selection of end-points and expression of results [24], so that comparison between the values reported by different laboratories is quite difficult [25]. Thanks to its simple and automatized protocol, PAOT-Liquid^®^ Technology overcomes these problems being operator independent.

Due to the strong correlation between antioxidant activity determined by the PAOT-Liquid^®^ Technology and TPC and using a calibration curve, winemakers could, therefore, be able to quickly monitor themselves if modifications in TPC content occur or not from grape harvest until wine bottling and storage. At least, another advantage of the PAOT-Liquid^®^ Technology is its moderate cost (around 10 €) when compared to more expensive tests performed in specialized laboratory analysis. Of interest is to note that the PAOT-Liquid^®^ Technology can also be used for determining antioxidant capacity of other types of non-alcoholic beverages, such as orange juices or plant extracts, as already described by us [26].

## Figures and Tables

**Table 1:** Characteristics of tested wines bought in a Belgian commercial market.

Number	Color	Region/Country	Name	Vintage	Year
1	red	Beaujolais/France	Moulin à vent	Gamay	2015
2	red	Cachapoal Valley/Chili	La Capitana	Merlot	2014
3	red	Bordeaux/France	Château Tuilerie Pages	Cabernet Franc, Merlot, Cabernet Sauvignon	2014
4	red	Bordeaux/France	Château la Tuilerie Graves	Merlot, Cabernet Sauvignon	2016
5	red	Corbières/France	Château Prat de Cest	Syrah, Grenache, Mourvedre	2015
6	red	Barossa Valley/South Australia	Lindeman’s Bin 50	Shiraz	2017
7	red	Mendoza/Argentina	Trivento	Malbec	2017
8	red	Bardolino/Italy	Giovanni Righetti	Corvina, Rondinella, Molinari	2017
9	red	Saint-Chinian/France	Valdorb rouge	Syrah, Grenache, Carignan	2017
10	red	Colchagua Valley/Chili	Koyle Reserva	Cabernet Sauvignon	2014
11	red	Western Cape/South Africa	Baie Cap	Pinotage	2017
12	red	Bourgogne/France	La chance du Roy	Gamay, Pinot Noir	2015
13	red	Minervois, France	L’aigle de Minerve	Carignan, Syrah, Grenache, Mourvedre	2016
14	red	Côtes du Rhône Villages/France	Côtes du Rhône villages	Grenache/Syrah	2016
15	rosé	Pays d’Oc/France	Syrah Rosé	Syrah rosé	2016
16	rosé	Pays d’Oc/France	Vin Gris	Cinsault, Syrah, Carignan, Grenache	2017
17	rosé	Corse/France	La Petite Paillote	Niellucciu, Sciaccarellu	2017
18	white	Pays d’Oc/France	Vent Marin	Chardonnay	2016
19	white	Val de Loire/France	Sauvignon de Touraine	Sauvignon Blanc	2017
20	white	Corse/France	La petite Paillote	Vermentino	2017

**Table 2:** PAOT-Liquid^®^ activity of several flavonoids, the major subclass of polyphenols family. Comparison with Trolox used as reference antioxidant in the DPPH assay.

	PAOT-Liquid^®^ Assay mg (GAE) L^−1^
Flavano-3-ol Family	
Catechin	504.56 ± 45.58
Epicatechin (EC)	730.2 ± 93.73
Gallocatechin (GC)	431.05 ± 35.61
Epigallocatechin (EGC)	545.58 ± 45.87
Epigallocatechin gallate (EGCG)	613.11 ± 0.57
Flavonol Family	
Kaempferol	404.56 ± 55.27
Quercetin	560.4 ± 0.85
Myricetin	677.78 ± 7.41
Flavanone Family	
Hesperdin methyl chalcone	51.85 ± 0.57
Naringin	53.28 ± 0.28
Anthocyanidins Family	
Pelargonidin Chloride	284.33 ± 3.42
Delphinidin Chloride	340.74 ± 69.23
Cyanidin Chloride	512.54 ± 5.13
Other	
Trolox	544.16 ± 16.81

**Table 3:** Total polyphenol content (TPC) in tested wines and their antioxidant capacity as assessed by DPPH method and PAOT-Liquid^®^ Technology.

Number	Region/Country	TPC mg (GAE) L^−1^	DPPH Assay µM (TE) L^−1^	PAOT-Liquid^®^ Assay mg (GAE) mg L^−1^
Red wines				
1	Beaujolais/France	2129 ± 17.9	3119 ± 47.7	1067.5 ± 17.86
2	Cachapoal Valley/Chili	1545 ± 40.1	2628 ± 24.9	908.02 ± 39.13
3	Bordeaux/France	2349 ± 18.2	3732 ± 32.6	1267.39 ± 30.2
4	Bordeaux/France	2253 ± 9.7	3773 ± 72.9	1180.21 ± 2.98
5	Corbières/France	1450 ± 20.3	2738 ± 65.3	757.03 ± 11.91
6	Barossa Valley/South Australia	1323 ± 12.8	3082 ± 51.3	1054.74 ± 17.86
7	Mendoza/Argentina	1603 ± 14.68	3168 ± 32.7	878.24 ± 26.79
8	Bardolino/Italy	1511 ± 11.8	1474 ± 11.0	846.35 ± 17.86
9	Saint-Chinian/France	1563 ± 24.9	2874 ± 44.8	950.55 ± 26.79
10	Colchagua Valley/Chili	2239 ± 20.8	4219 ± 64.6	1280.15 ± 6.34
11	Western Cape/South Africa	1915 ± 17.5	2395 ± 20.1	942.04 ± 2.98
12	Bourgogne/France	1278 ± 41.5	1240 ± 4.5	1086.64 ± 8.93
13	Minervois, France	2060 ± 8.8	3912 ± 63.5	959.05 ± 32.75
14	Côtes du Rhône Villages/France	1831 ± 37.8	3065 ± 57.2	1088.77 ± 17.86
mean		1789	2958	1016.47
SD		367	854	153.11
